# Structural and functional insight into the universal stress protein family

**DOI:** 10.1111/eva.12057

**Published:** 2013-03-13

**Authors:** Karolina L Tkaczuk, Igor A Shumilin, Maksymilian Chruszcz, Elena Evdokimova, Alexei Savchenko, Wladek Minor

**Affiliations:** 1 Department of Molecular Physiology and Biological Physics, University of VirginiaCharlottesville, VA, USA; 2 Midwest Center for Structural GenomicsUSA; 3 Department of Chemical Engineering and Applied Chemistry, University of TorontoToronto, ON, Canada; 4 Department of Chemistry and Biochemistry, University of South CarolinaColumbia, SC, USA

**Keywords:** *Archaeoglobus fulgidus*, crystal structures, *Nitrosomonas europaea*, pathogens, sequence analyses, structural comparison, structural genomics, universal stress protein

## Abstract

We present the crystal structures of two universal stress proteins (USP) from *Archaeoglobus fulgidus* and *Nitrosomonas europaea* in both *apo*- and ligand-bound forms. This work is the first complete synthesis of the structural properties of 26 USP available in the Protein Data Bank, over 75% of which were determined by structure genomics centers with no additional information provided. The results of bioinformatic analyses of all available USP structures and their sequence homologs revealed that these two new USP structures share overall structural similarity with structures of USPs previously determined. Clustering and cladogram analyses, however, show how they diverge from other members of the USP superfamily and show greater similarity to USPs from organisms inhabiting extreme environments. We compared them with other archaeal and bacterial USPs and discuss their similarities and differences in context of structure, sequential motifs, and potential function. We also attempted to group all analyzed USPs into families, so that assignment of the potential function to those with no experimental data available would be possible by extrapolation.

## Introduction

Universal stress proteins (USP) are widely spread proteins in nature. In the Pfam classification, USPs belong to the PF00582 superfamily (COG0589) (Tatusov et al. 2003; Bateman et al. 2004) and are present in a diverse set of organisms from archaea and bacteria to fungi and plants. This evolutionary abundance shows their importance for all three domains of the tree of life taxonomy. In stress conditions such as heat shock, nutrient starvation, the presence of oxidants, uncouplers, DNA-damaging agents, or other stress agents which may arrest cell growth, USP constitute a natural biological defense mechanism. Under stress, USPs are overproduced and through a variety of mechanisms aid the organism in surviving in such uncomfortable conditions. It is also predicted that USPs are helping pathogens, that is, *Salmonella*, *Klebsiella*, or *Mycobacterium*, in invasion of the host organisms (Rayan and Ray 2004; Hensel 2009), which presents potential new opportunities for the pathogenic infection treatment. The studies by Hingley-Wilson et al. (2010) suggest involvement of *usp* genes in the persistence or/and intracellular survival of *Mycobacterium tuberculosis*. Similarly Liu et al. showed that USPs play a significant role in *Salmonella* growth arrest, stress, and virulence (Liu et al. 2007).

**Figure 1:**
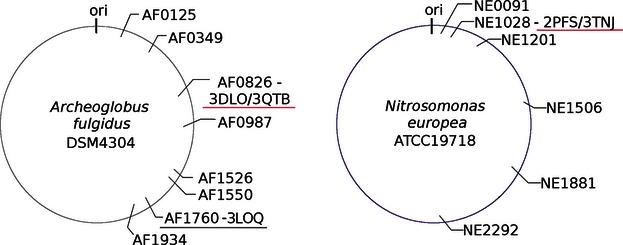
Schematic maps of the *Archeoglobus fulgidus* and *Nitrosomonas europea* chromosomes, showing the positions of *usp* genes. The names on the map are *usp* genes labeled by locus tag. If the structure of the protein encoded by an *usp* gene has been determined, the gene is labeled with the Protein Data Bank codes and underlined in red.

Most organisms have multiple paralogs of USPs, where the number of copies depends on the organism. In *Escherichia coli*, there are six USPs (UspA, UspC (*yecG*), UspD (*yiiT*), UspE (*ydaA*), UspF (*ynaF*), and UspG (*ybdQ*), where UspE is a fusion protein composed of two USP units E1 and E2). In *Arabidopsis thaliana*, for instance, there are four copies of *usp* genes. In *Nitrosomonas europea* and *Archaeoglobus fulgidus* investigated in this study in more detail, there are six and eight known copies of genes encoding for USPs, respectively (Fig. 1). The exact function of these proteins is unknown or there are very little details that can help decipher their role in aforementioned cellular processes. There are multiple copies of USP proteins that are not assigned to any of the above-mentioned groups (UspA-F); thus it is extremely difficult to even try to predict the type of the process they can be associated with. Such assignment would be especially helpful in case of medically relevant organisms, like pathogens, that is, *M. tuberculosis* (causing TB), *Klebsiella pneumonia* (opportunistic pathogen causing pneumonia in form of bronchitis), *Salmonella enterica* (responsible for salmonellosis), or *Burkholderia* genus (causative factor of melioidosis or/and cystic fibrosis).

**Figure 2:**
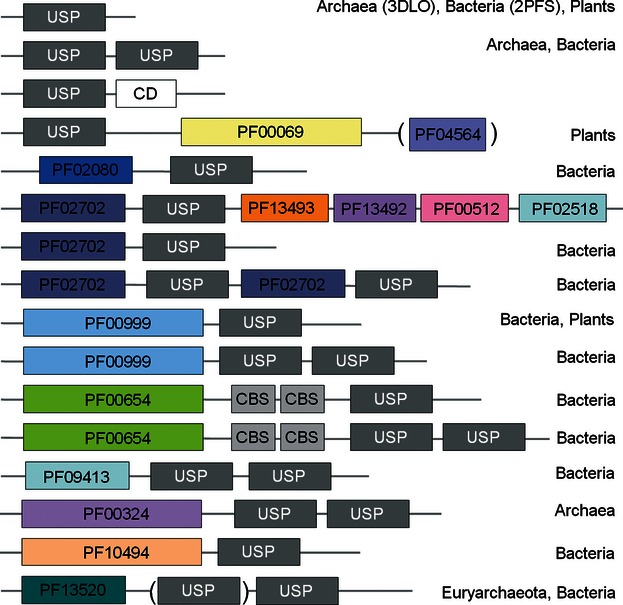
Domain composition of known USP proteins. In this figure, USP denotes the universal stress protein domain, CBS is the cystathionine beta synthase domain, and CD is a conserved domain of unknown function. All other domains are labeled by Pfam family identifier: PF00069 is a protein kinase domain, PF04564 the U-box domain, PF02080 the TrkA domain (unknown function), PF02702 the KdpD domain, PF13493 the DUF4118 domain (unknown function), PF00512 the HisKA domain, PF02518 the *HATPase_c* domain, PF00999 the sodium/hydrogen exchanger family domain, PF00654 the Voltage CLC domain, PF09413 the DUF2007 domain (unknown function), PF10494 the Stk19 domain (a family of Ser/Thr protein kinases), and PF13520 the *AA_permease_2* domain (a family of amino acid permeases).

Universal stress proteins occur both as single-domain proteins and fusions with extra domains, where the extra domain may be an additional USP domain as in the case of protein PA1789 from *Pseudomonas aeruginosa* POA1, a protein kinase domain in case of some plants, or an amino acid permease followed by two USP domains in some archaea (Fig. 2). USPs can be divided into those that bind ATP (UspFG-type) and those that do not (UspAs and UspA-like group) (Kvint et al. 2003).

Currently, there are 26 redundant USPs crystal structures available in the Protein Data Bank (PDB) (Table 1). This set comprises 21 structures of bacterial USPs (two from *Nitrosomonas europaea*, one from *Proteus mirabilis*, one from *Halomonas elongata*, six from *Thermus thermophilus* HB8, one from *P. aeruginosa* PAO1, two from *K. pneumoniae*, two from *Lactobacillus plantarum* WCFS1, three from *M. tuberculosis*, one from *Wolinella succinogenes*, one from *Aquifex aeolicus,* and one from *Haemophylus influenzae*), four structures from archaea (one from *Methanococcus jannashi* and three from *A. fulgidus*), and one structure from a eukaryote (the plant *A. thaliana*). The best-studied family members are MJ0577 from *M. jannashi* (Zarembinski et al. 1998), UspA from *H. influenzae* (Sousa and McKay 2001), and *E. coli* (Nystrom and Neidhardt 1992). In *E. coli*, UspA does not contain an ATP-binding site, while *E. coli* UspF binds ATP. The differences between UspA and UspF in the binding of ATP, despite their significant structural similarity, suggest that the UspA and UspFG subgroups typified by these two *E. coli* proteins display different substrate specificities (Weber and Jung 2006).

**Table 1:** General information on USP crystal structures deposited in the Protein Data Bank (PDB)

Name	PDB code	Organisms	Annot.	ATP-rel.	ATP-binding motif	Motif	ATP	Ion	SG center	Ref.
NE1028	2PFS/3TNJ	*N. europea*	USP	D12, V40	G^114^-SH-G^117^-(8X)-G^126^ST	Typical	No/AMP	No	MCSG	–*
AF0836	3DLO/3QTB	*A. fulgidus*	USP	D11, S40	G^103^-IR-K^106^ -(9X)-G^116^SV	Typical	No/dAMP	Cl	MCSG	–3
AF1760	3LOQ	*A. fulgidus*	USP	D154,V182	G^234^-SR-G^237^-(9X)-G^247^ST	Typical	AMP	Cl	MCSG	n/a
Lp1163	3S3T	*L. plantarum*	USP	D13, V41	G^115^-AT-G^118^-(9X)-G^128^ST	Typical	ATP	Ca	MCSG	n/a
Lp3663	3FG9	*L. plantarum*	UspA	D20, V50	G^123^-AD-T^126^-(11X)-G^138^PR	Degen.	No	Mg	MCSG	n/a
WS0661	3IDF	*W. succinogenes*	USP	D9, V38	G^108^-SS-E^111^-(8X)-A^120^SH	Degen.	No	No	MCSG	n/a
Rv2623	3CIS	*M. tuberculosis*	UspE	D167,A195	G^262^-SR-G^265^-(9X)-G^275^SV	Typical	ATP	Mg	MCSG	n/a
Rv1636	1TQ8	*M. tuberculosis*		D25, A52	G^126^-NV-G^129^-(9X)-G^139^SV	Typical	No	No	NYSGXRC	n/a
Rv2623	2JAX	*M. tuberculosis*	UspE	D167,A195	G^262^-SR-G^265^-(9X)-G^275^SV	Typical	ATP	No	n/a	n/a
KPN01444	3FH0	*Klebsiella pneumoniae*	USP	D7, V37	A^111^-SH-R^114^-(8X)-G^123^SN	Degen.	ADP	No	MCSG	n/a
KPN01444	3FDX	*K. pneumoniae*	UspF	D7, V37	A^111^-SH-R^114^-(8X)-G^123^SN	Degen.	ATP	Mg	MCSG	n/a
AT3G01520	2GM3	*A. thaliana*	USP	N13, V53	G^131^-SR-G^134^-(9X)-G^275^SV	Typical	AMP	No	CESG	n/a
Aq178	1Q77	*A. aeolicus*	UspA	D9, V37	A^113^-CY-P^130^	Degen.	No	No	MCSG	n/a
PMI1202	3OLQ	*P. mirabilis*	UspE	N161,A198	G^270^-IL-G^273^-(10X)-N^284^TA	Degen.	No	No	MCSG	n/a
PA1789	3MT0†	*Pseudomonas aeruginosa*	UspE	D139,A174	G^241^-TV-A^244^-(9X)-G^254^NT	Degen.	No	Cl	MCSG	n/a
	1WJG	*T. thermophilus*HB8	–‡	D10, A38	G^106^-TR-G^109^-(9X)-G^119^SQ	Typical	No	No	RSGI	n/a
TTHA0895	2Z3V	*T*. *thermophilus*HB8	USP	D10, A38	G^106^-TR-G^109^-(9X)-G^119^SQ	Typical	No	No	RSGI	n/a
TTHA0895	2Z08/9	*T*. *thermophilus*HB8	USP	D10, A38	G^106^-TR-G^109^-(9X)-G^119^SQ	Typical	ATP/ACT	Mg	RSGI	n/a
TTH0350	3AB7/8	*T*. *thermophilus* HB8	USP	D8, V36	G^121^-RS-D^124^-(5X)-G^130^ST	Degen.	ATP	Mg	n/a	(1)
HI0815	1JMV	*H. influenzae*	UspA	D10, A38	G^109^-HH-Q^112^-(6X)-M^119^SV	Degen.	No	No	n/a	(2)
HELO1754	3HGM	*H. elongata*	TeaD	D10, A38	G^117^-AE-G^120^-(9X)-G^130^SV	Degen.	ATP	Mn	n/a	(3)
MJ0577	1MJH	*M. jannaschii*	–‡	D13, V41	G^127^-SH-G^130^-(9X)-G^140^SV	Typical	ATP	Mn	BSGC	(4)

MCSG, Midwest Center for Structural Genomics; RSGI, RIKEN Structural Genomics/Proteomics Initiative; CESG, Center for Eukaryotic Structural Genomics; BSGC, Berkley Structural Genomics Center; NYSGXRC, New York SGX Research Consortium.

X denotes structures not solved by structural genomics centers.

* This work, X denotes any residue and the digit in front of it the number of X residues. AMP, ADP, and ACT are the following derivatives of ATP: ACT, phosphomethylphosphonic acid adenylate ester (C_11_H_18_N_5_O_13_P_3_); AMP, adenosine monophosphate (C_10_H_14_N_5_O_7_P); ADP, adenosine-5′-diphosphate (C_10_H_15_N_5_O_10_P_2_). (i) publication by Iino et al. (2011), (ii) work by Sousa and McKay (2001), (iii) publication by Schweikhard et al. (2010), (iv) work by Zarembinski et al. (1998).

† Special case of USP.

‡ ATP-binding protein.

Here, we present the structures of both *apo-* and cofactor-bound forms of an euryarchaeal USP AF0826 from *A. fulgidus* and a bacterial USP NE1028 from *N. europaea*. We also discuss the structural and sequential similarities and differences between them in the context of the entire USP superfamily based on the comprehensive sequence-structure analyses of all available 3D structures of USP proteins and clustering analysis aiming at their division into UspA-F groups. The three-dimensional structures of these USPs give valuable clues for the understanding of their potential biochemical mechanisms, although the precise biological functions of these proteins remain not known.

## Materials and methods

### Protein cloning, expression, purification, and crystallization

Both NE1028 from *N. europaea* and AF0826 from *A. fulgidus* containing N-terminal His_6_-tags followed by the tobacco etch virus (TEV) protease cleavage sites were cloned, expressed, and purified using previously described methods (Zhang et al. 2001). The His-tag of NE1028 was readily cleaved by TEV protease. Selenomethionine (SeMet)-substituted NE1028 was used to determine the *apo*-structure and the wild-type protein was used in the complex structure solution. TEV protease was not efficient in removing the His-tag from AF0826 and this protein was purified with the tag attached. SeMet-substituted AF0826 has been used for both *apo* and complex structures.

Crystals of SeMet-substituted *apo*-NE1028 were grown by the vapor diffusion method in a hanging drop at 293 K. Crystallization drops consisting of 1.5 μL of 10-mg/mL protein solution and 1.5 μL well solution [25% w/v polyethylene glycol (PEG) 3350, 0.2 m NaCl, 2% isopropanol, and 0.1 m HEPES pH 7.5] were equilibrated against 200 μL of well solution.

Crystals of wild-type NE1028 were grown by the vapor diffusion method in a hanging drop at 293 K. The crystallization drops consisted of 1.5 μL protein solution (10 mg/mL NE1028) and 1.5 μL well solution [46% v/v PEG 400, 10 mm adenosine-5′-monophosphate (AMP), 6% w/v xylitol, and 0.1 m Bis-Tris pH 7.0] and were equilibrated against 200 μL of well solution. The NE1028 crystals were soaked with 10 mm of AMP for 24 h.

Crystals of the SeMet-substituted AF0826 were grown by vapor diffusion method in a hanging drop at both 273 and 293 K. Crystallization drops containing 1.5 μL of 10-mg/mL protein solution and 1.5 μL well solution (25% w/v PEG 3350, 0.2 m ammonium acetate and 0.1 m Bis-Tris pH 5.5) were equilibrated against 200 μL of well solution. To obtain a complex of AF0826 with a ligand, the crystals were soaked with a metabolite cocktail that contained adenine, adenosine, AMP, adenosine-5′-diphosphate ADP, ATP, 2′-deoxyadenosine-5′-monophosphate dAMP, cAMP, and ADP-ribose. Subsequent structure solution identified the bound ligand as dAMP.

All crystals selected for data collection were transferred into paratone–N oil and flash cooled in liquid nitrogen at 100 K.

### Data collection, structure determination, and refinement

All X-ray diffraction data were collected at the Advanced Photon Source (APS) of the Argonne National Laboratory. Diffraction data for the *apo*-form of *A. fulgidus* AF0826 were collected at the 19–BM beamline (Rosenbaum et al. 2006). Diffraction data for the dAMP-bound form of AF0826 were collected at beamline 21ID-G. Diffraction data for the *N. europaea* protein (NE1028) were collected at the 19-ID beamline for crystals of the apo-form and the 19–BM beamline for crystals of AMP-bound NE1028 structures. All diffraction data were processed and scaled with the HKL–2000 program suite (Otwinowski and Minor 1997). The data collection statistics are summarized in Table 2. The structure of the *apo*-form of *N. europaea* protein was determined by single-wavelength anomalous dispersion (SAD), while *apo*-AF0826 from *A. fulgidus* was determined by molecular replacement (MR) based on three USP structures Rv1636 (1TQ8), Rv2623 (2JAX), and 1WJG. Both structures with ligands were determined by molecular replacement (MR), using the respective *apo*-form structures as the initial model. In all cases, initial phase calculations, electron density map modification, and initial model building were done using the HKL–3000 program (Minor et al. 2006). The HKL–3000 software package interacts with SHELXD, SHELXE (Sheldrick 2008), MLPHARE (Otwinowski 1991), DM (Cowtan and Main 1993; Cowtan and Zhang 1999), CCP4 (Winn et al. 2011), SOLVE (Terwilliger and Berendzen 1999), RESOLVE (Terwilliger and Berendzen 1999; Terwilliger 2002), ARP/wARP (Perrakis et al. 1999), O (Jones et al. 1991), and COOT (Emsley and Cowtan 2004). After the initial models were built, the remaining parts of the models were extended manually with COOT. The models, were then refined using REFMAC5 (Murshudov et al. 2011). Solvent atoms were initially built with ARP/wARP, and solvent atoms were later manually added or removed as needed. Models (and the experimental structure factors) were assessed and validated by SFCHECK (Vaguine et al. 1999), PROCHECK (Laskowski et al. 1993), ADIT (Yang et al. 2004), MOLPROBITY, and KING (Lovell et al. 2003).

**Table 2:** Data collection and structure determination statistics. Crystallographic parameters, data-collection (native data) and refinement statistics for *Archaeoglobus fulgidus* and *Nitrosomonas europaea* proteins (apo and ligand-bound structures)

	apo-AF0826 3DLO	AF0826-dAMP 3QTB	apo-NE1028 2PFS	NE1028-AMP 3TNJ
Data collection				
Beamline	19-BM	21-ID	19-ID	19-BM
Wavelength (Å)	0.9793	0.9792	0.9792	0.9791
Resolution (Å)	1.97 (1.97–2.03)	2.10 (2.10–2.14)	2.25 (2.25–2.29)	2.00 (2.00–2.03)
Space group	P2_1_	C2	P32_1_	P32_1_
a (Å)/b (Å)	43.2/99.2	109.6/42.7	76.0/77.8	77.8/77.8
c (Å)	57.4	61.3	43.0	39.9
α/β (°)	90.0/92.4	90.0/116.8	90.0/90.0	90.0/90.0
γ (°)	90.0	90.0	120.0	120.0
Solvent content (%)	30.7	33.3	42.5	41.5
Completeness (%)	99.6 (77.6)	99.6 (98.8)	97.60 (81.5)	97.5 (93.7)
Observed reflections	33270	36964	6882	9070
Unique reflections	33213	33270	6538	8637
I/σ (I)	24.1 (2.7)	21.5 (2.6)	58.5 (2.9)	29.0 (2.7)
R_merge_ (%)	7.3 (40.5)	7.0 (40.5)	6.0 (48.6)	5.1 (57.5)
Refinement				
R (%)/R_merge_ (%)	17.5/23.0	20.0/23.7	19.8/25.5	19.1/24.1
Mean *B* values (Å^2^)	23.2	38.9	52.4	48.8
Protein atoms	4398	1970	944	996
Chloride ions	2	0	2	0
Water molecules	182	50	40	36
Structure quality				
Ramachandran statistics*				
Favored (%)/*n*	97.9	99.6	100	100
Allowed (%)/*n*	2.1	0.4	0	0
All-atoms contacts and protein geometry				
Clash score	12.25 (71st)†	8.15 (93rd)†	9.17 (93rd)†	7.47 (93rd)†
MolProbity score	2.13 (63rd)†	1.44 (99rd†)	1.85 (93rd)†	1.61 (94rd)†
RMS deviation				
Bond lengths (Å)	0.019	0.013	0.017	0.014
Bons angles (°)	1.8	1.5	1.6	1.4

Data for the highest resolution shell are given in parentheses.

* Pro and Gly residues were excluded from calculation.

* Percentile.

The atomic coordinates and structure factors for the four models have been deposited in the PDB. The accession codes for the NE1028 structures are 2PFS (*apo*-form) and 3TNJ (AMP-bound form), and for AF0826 are 3DLO (*apo*-form) and 3QTB (dAMP-bound form).

### Sequence searches and analyses

Sequence searches were carried out using PSI–BLAST (Altschul et al. 1997), and multiple sequence alignments were constructed using MUSCLE (Edgar 2004) from a nonredundant sequence set. The conserved domain search tool (CD) was used to predict duplication of domains within the analyzed data set, as compared against the conserved domain database (CDD) (Marchler-Bauer and Bryant 2004; Marchler-Bauer et al. 2009).

### Sequence clustering

To visualize pairwise similarities between and within protein families, we used CLANS (CLuster ANalysis of Sequences), a Java utility that implements a version of the Fruchterman–Rheingold graph layout algorithm (Frickey and Lupas 2004). A three-dimensional representation of the similarity graph is built by randomly seeding nodes representing each sequence in space. The sequences nodes are iteratively moved within this environment by applying all force vectors to each node that are (i) proportional in amplitude to the similarity between each pair of sequences and (ii) in the direction of the edge connecting each pair of nodes. This process is continued until the overall shape of the graph converges.

### Homology modeling

The missing loops (residues N45–T55 and G119–G128) in the 2PFS structure were modeled by homology modeling. The hybrid model was constructed using the ‘FRankenstein's Monster’ approach (Kosinski et al. 2003), comprising cycles of model building using MODELLER (Fiser and Sali 2003) and SWISS-Model (Schwede et al. 2003), followed by evaluation using Verify3D (Luthy et al. 1992).

### Evolutionary history

The evolutionary history of members of the USP superfamily was inferred using the Neighbor-Joining method (Saitou and Nei 1987). The bootstrap consensus tree was inferred from 500 replicates and represents the evolutionary history of the taxa analyzed (Felsenstein 1985). Branches corresponding to partitions reproduced in <50% of the bootstrap replicates were collapsed. The percentage of replicate trees in which the associated taxa clustered together in the bootstrap test (500 replicates) are shown next to the branches (Felsenstein 1985). The tree is drawn to scale, with branch lengths in the same units as those of the evolutionary distances used to infer the phylogenetic tree. The evolutionary distances were computed using the JTT-matrix-based method (Jones et al. 1992) and are in units of number of amino acid substitutions per site. The data used for analysis comprised 176 amino acid sequences. All ambiguous positions were removed for each sequence pair. There were a total of 224 positions in the final data set. The evolutionary analyses were conducted using the MEGA5 program (Tamura et al. 2011).

### Structure analysis

Sequence conservation was calculated from the sequence alignment and mapped onto the protein structure using ConSurf (Armon et al. 2001; Glaser et al. 2003). Structures were manipulated and modeled using SwissPDBViewer (Guex and Peitsch 1997), and visualizations and structure figures were generated using PyMol (DeLano 2002). Database searches by structural similarity and structure superimpositions were done using DALI (Holm and Sander 1993). Dimerization patterns were predicted using a 3D-structure-based, not sequence-based, predictor called meta-PPISP (Qin and Zhou 2007). Biological assemblies were predicted using PISA (Krissinel 2010).

## Results and discussion

### Structures of USPs from *Nitrosomonas europaea* and *Archaeoglobus fulgidus*

The structure of the *apo*-form of NE1028, a β-proteobacterial USP from *N. europaea* was refined to a final resolution of 2.25 Å (Table 2). There was no interpretable electron density for the two loops comprising residues N45–T55 and G119–G128, perhaps due to high mobility. These loops include the potential ATP-binding residues, so they were reconstructed using homology modeling. The new model of the full-length protein (including the modeled loops) was used for purposes of comparative analysis.

**Figure 3:**
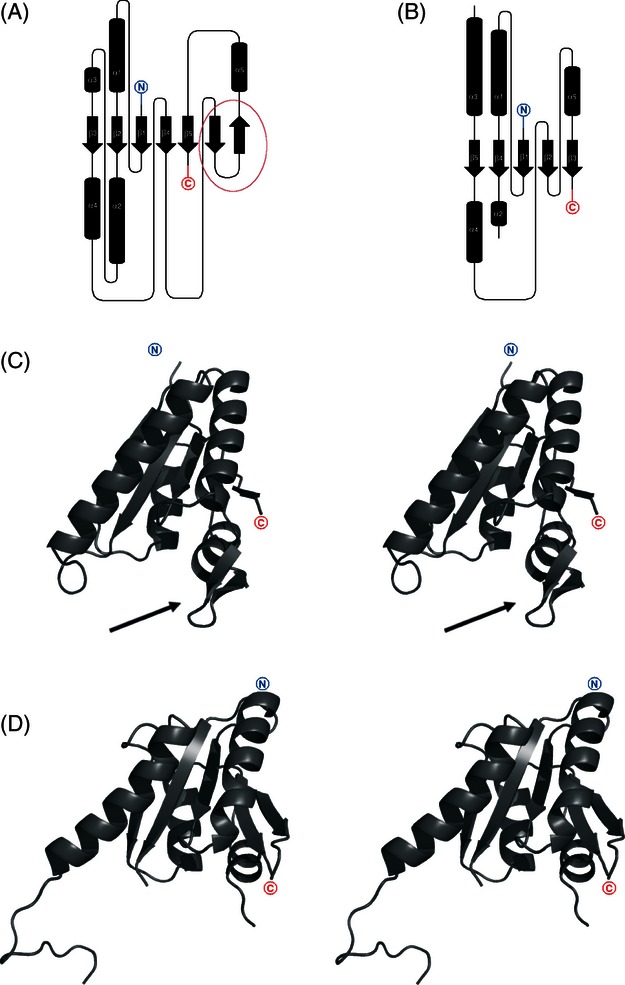
Topology key note diagrams of (A) AF0826 and (B) NE1028 structures. Cylinders represent α-helices while arrows correspond to β-strands (C) cartoon representation of AF0826 monomer; the arrow shows the location of the b-hairpin insertion (D) cartoon representation of NE1028 monomer.

The monomer structure of *apo*-NE1028 is an open, twisted, five-strand parallel β-sheet with topology β3-β2-β1-β4-β5, sandwiched by α-helices (Fig. 3). It strongly resembles the structure of the MJ0577 protein from *M. jannashii* (PDB code: 1MJH), which was determined with ATP bound between β1 and β4 and previously postulated by Zarembinski and coworkers (Zarembinski et al. 1998) to be an USP-type protein and became a model USP protein.

The structure of the *apo* form of AF0826, a euryarchaeal USP from *A. fulgidus* was refined to a final resolution of 1.97 Å (Table 2). The monomer structure is very similar to the structure of previously described NE1028 USP. It also forms an open, twisted, five-strand parallel β-sheet, although in AF0826, two additional β-strands forming a β-hairpin structural motif are inserted between β4 and β5 (Fig. 3A). For ease of comparison, sequentially analogous β-strands in both NE1028 and AF0826 are numbered identically. In all other known USP structures, the residues found between strands β4 and β5 region form a loop. The topology of the β-sheets in both USPs presented in this work (archaeal and proteobacterial) are the same.

Structure solution and refinement statistics for all structures are given in Table 2.

### Bioinformatics studies

To provide an additional functional insight, we collected USP sequences from the NCBI database as well as all USP structures available in the PDB. After redundant sequences were removed, the resulting data set was used for a series of sequential and structural comparisons. This comprehensive analysis was aimed at comparing potential ligand-binding sites, metal ion contribution, dimerization patterns, and the evolutionary relatedness of NE1028 and AF0826 to previously characterized members of the superfamily. We have done a clustering analysis of previously assigned USP proteins. The results of these analyses are presented in detail below.

### Ligand-binding sites in USP proteins

**Figure 4:**
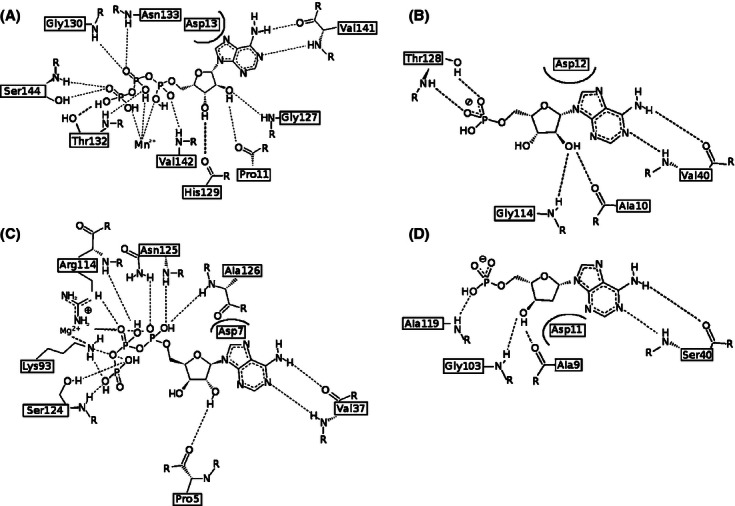
Ligand-binding sites of USP family members. (A) ATP-binding residues in the best characterized USP, namely UspA MJ0577 from *M. jannaschii*; (B) universal stress protein NE1028-AMP from β-proteobacterium *N. europea*; (C) fusion UspE protein KPN01444-ATP from *Klebsiella pneumonia*; (D) universal stress protein AF0826-dAMP from euryarchaeota *Archeoglobus fulgidus*.

Known structures of USP monomers were superimposed by DALI and analyzed. Subsequently, potential ligand (ATP)-binding sites were identified in each structure. Potential ATP-binding residues were defined on the basis of ATP-binding motif described previously in the literature (O'Toole and Williams 2003), which are shown in Table 1 and Fig. 4. This analysis shows that for USPs with typical ATP-binding motifs, almost all structures were solved with ATP or an ATP analog, while for USPs, where this motif is completely degenerated, neither ligand nor ion binding was observed. As this is a correlation of existing data rather than the results of a controlled experiment, it is impossible to determine whether the absence or presence of ligand binding is due to the corresponding absence or presence of the crucial residues, or this correlation is due to other factors (e.g., whether or not cocrystallization was done).

The common motif in determined structures of USPs that have been experimentally proven to bind ATP is G-(2X)-G-(9X)-G(S/T). This is similar to the Walker A motif and it is composed of residues located between α1, β1, β2, and β4. Our analysis shows that for the conserved residues of the motif: the first Gly is present in 81% of the structures (21 of 26), the second Gly in only 50%, the final Gly in 80%, and the final S or T in 81% of all structures, respectively. ATP-binding motifs in experimentally determined USP structures are presented in the Table 1.

The best-studied USP protein MJ0577-ATP from *M. janashii* contains a typical ATP-binding motif, which is composed of the following residues: G^127^-SH-G^130^-(9X)-G^140^S^141^V^142^ (where the superscripts indicate the position of each residue in the sequence, Fig. 4A). This motif corresponds to the following residues in NE1028: G^114^-SH-G^117^-(8X)-G^126^S^127^T^128^, suggesting that the *N. europea* USP may be able to bind ATP. Our structure of NE1028–AMP confirms that the protein can bind at least one molecule of ATP analog per monomer (Fig. 4B). In the NE1028–AMP structure, a conserved stretch of nonpolar residues (as visible on summarizing multiple sequence alignment—Fig. 8), followed by D^14^ and also strongly conserved S/T residues and V^42^, form contacts with the adenine ring. The first three residues from the conserved motif (G^116^-SH-) contacts ribose and the T^130^ residue binds the phosphates.

Figure 4 shows the arrangement of amino acids potentially (or confirmed to be) involved in the ATP/its analog binding in different representatives of USP superfamily. *apo*-AF0826 contains a nearly canonical ATP-binding motif (G^103^-IR-K^106^-(9X)-G^116^S^117^V^118^). The only difference is that it lacks the second Gly residue which is replaced by Lys (K^106^), the spatial arrangement of aforementioned residues creates nearly perfect environment for the binding of ATP or one of its derivatives. Based on this observation, we hypothesized that soaking or cocrystallization of that protein with ATP could result in formation of a crystal with the ligand bound in this region. As a result, we obtained the structure of AF0826 with dAMP bound in the vicinity of the ATP-binding motif (Fig. 4D). All of the residues binding dAMP in AF0826 are analogs of the residues binding AMP in the NE1028 structure, and interact with the ligand in very similar ways, as shown in Fig. 4B and D. Where hydroxyl groups are interacting with G and A (AF0826: G103, A9; NE1028: G114, A10) and adenine ring is positioned by either V or S (S40 and V40, respectively). These observations are in perfect agreement with the observations made by Iino and coworkers (Iino et al. 2011) for TTHA0350 from *Thermus thermophiles* HB8 (PDB: 3AF7/8). They also extrapolated their predictions onto AF0826 claiming that the cavity of AF0826, formed by aforementioned residues, may accommodate the adenosine part of ATP, which indeed turned out to be true.

### Ion-water-mediated coordination of ATP molecules in USPs

Data presenting potential ATP/ADP/AMP-binding motifs and metal ions bound in the structure for all USPs of solved structure are summarized in Table 1. The consensus ATP-binding motif is present in UspE domains 1 and 2 of Rv2623 (3CIS), MJ0577 (1MJH), TTHA0895 (2Z3V), and AT3G01520 (2GM3), which have been previously shown to bind ATP (AMP for AT3G01520). Most but not all structures crystallized to date were solved with divalent metal ions such as manganese or magnesium. In the case of protein MJ0577, the ATP molecule is positioned by the octahedral coordination of a manganese ion, at least three water molecules, and several protein residues (Zarembinski et al. 1998; Schweikhard et al. 2010). In contrast, the apo-AF0826 and AF1760-AMP structures were determined to contain chloride ions, which are not located in the same place as the divalent ions, while AF1760 still contains the ligand molecule. Therefore ion/water-mediated coordination of ATP molecules in USP may not be present in all cases. This mechanism could be function related and present only in certain USP families. The chloride ions present in both structures may come from the crystallization solution or cell and play some other role if any.

In 2001, Sousa and McKay (Sousa and McKay 2001) had proposed that members of the USP superfamily can be divided into two groups by whether or not they bind ATP, as this could explain their divergence toward different biological functions. Interestingly, as pointed out later by Schweikhard and coworkers on the example of the USP from *K. pneumonia* (KPN01444-ADP – 3FH0 and KPN01444-ATP – 3FDX), the lack of a (fully) conserved ATP-binding motif does not necessarily preclude classifying a given USP as non-ATP-binding (Schweikhard et al. 2010). The *K. pneumonia* USP they studied has a partially degenerated ATP-binding motif (see Table 1). The same is true for a USP HELO4277 from *H. elongate* (PDB code; 3HGM) in which case both ATP and magnesium ion are shown to be bound in its structure. In case of a USP from *A. aeolicus* where the ATP-binding motif is completely degenerated, neither metal ion binding nor ligand binding was observed in its structure. There are also a few cases shown in Table 1 where a typical or nearly typical ATP-binding motif is observed, but no additional molecules are observed in the crystal structures. (There are couple of potential explanations for that (i) an absence of the ligand molecules in the crystallization conditions, (ii) soaking with ATP experiments were not performed or unsuccessful, (iii) these USP simply do not bind ATP.) Finally, the tandem USP TTHA0350 from *T. thermophilus* HB8 contains a conserved ATP-binding motif in its N-terminal USP domain and a completely degenerated ligand-binding motif in the C-terminal domain, but still binds two ATP molecules, one on each side of the ‘tandem’ monomer.

### Dimerization patterns of USP proteins

**Figure 5:**
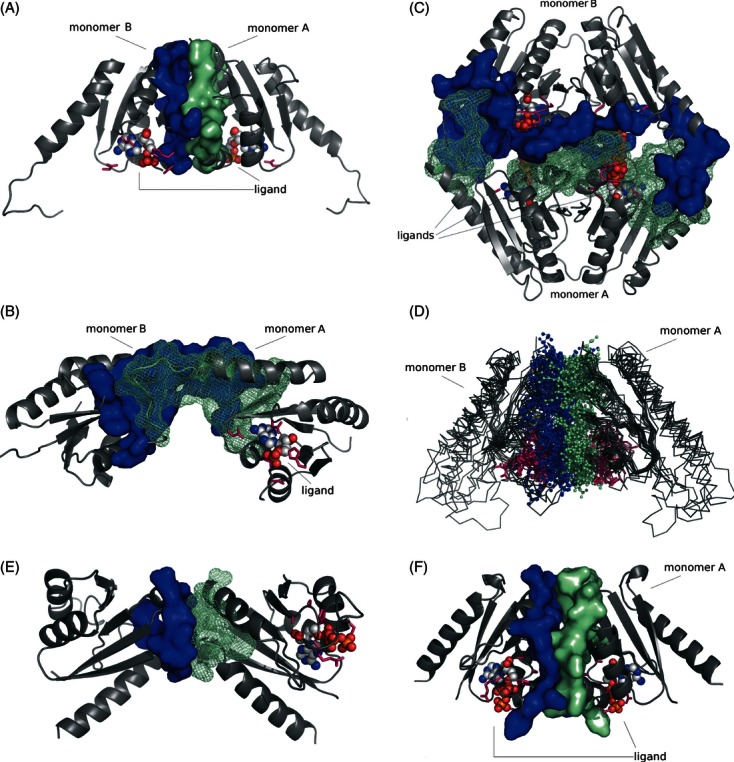
Dimerization pattern of USP family members. (A) probable dimer assembly of USP NE1028 from *N. europea* [Protein Data Bank (PDB) code: 2PFS]; (B) likely incorrect dimeric assembly of USP NE1028 from *N. europea* (PDB code: 2PFS) predicted by the PISA server; (C) dimeric assembly of UspE protein Rv2623 from *Mycobacterium tuberculosis* (PDB code: 3CIS); (D) superposition of type 1 dimers (representatives listed in the Table 1); (E) Incorrect UspF assembly (PISA AB); (F) Correct assembly (PISA AA) of UspF (PDB code: 3FDX) ATP-binding residues are shown in pink, dimerization interface residues from monomers A and B are shown in green and blue respectively, and ligand molecules are shown in CPK colors in either space-filling or ball-and-stick representation.

As previously demonstrated in the literature, on the example of MJ0577 and HI0815, USP proteins form stable homodimers (Zarembinski et al. 1998; Sousa and McKay 2001; Weber and Jung 2006), the dimeric state of AF0826 and NE1028 was verified by size-exclusion chromatography and dynamic light scattering (data not shown). There are two main patterns of dimerization predicted for known USP structures. Most USPs appear to dimerize in the same way as the protein from *N. aeruginosa* (PDB code: 2PFS) as shown in Fig. 5A, where the dimer interface is formed largely by the C-terminus of each monomer (henceforth called type 1). This type of dimerization is formed via interactions of strongly hydrophobic, structurally and sequentially conserved β-strands: β5 and β5′ (where the prime on β5′ denotes the β5 strand from another monomer) and α4 and α4′, followed by loops joining β4 and α4 and β4′ and α4′, which are less conserved and in some cases adopt configurations with partial secondary structure elements (α-helix or β-strand). AF0826 displays the same type 1 dimerization, the dimerization interface is formed by β5 and α4 from each monomer as it takes place is case of NE1028. Type 1 dimers superimpose very well as presented on the Fig.D.

Our observations show that one should pay a lot of attention when defining biological assemblies for crystal structures. The first biological assembly we defined for *apo*-NE1028 from *N. aeruginosa* without taking into account any potential evolutionary/similarity information led to a likely misassignment of the dimer interface. Determination of the dimerization state with the PISA produced the dimer shown on the Fig. 5B. In this case, the (probably inaccurately) modeled dimer appears to be formed by the long α2 helix from each monomer and loops adjacent to it, which are both located in the middle of the protein structure. In this variant, ATP-binding sites are located on the outside of each monomer, so if they would form tetramer the ligand-binding site would be located on the dimerization interface making the cavity unavailable for ATP. This dimerization solution scored as potentially the best one according to the PISA server, with a Complexation Significance Score (CSS) of 0.254, and a decrease in solvent-accessible surface area (ASA) of 955 Å^2^. However, after we took a closer look at the dimerization pattern seen for other members of the USP family, especially the UspE proteins (Fig. 5C, described below), it became obvious that this is probably an incorrect assembly. The connecting loops indeed take part in the dimerization of UspE; however, the orientation of particular monomers is different. This mistake was noticed and corrected when we analyzed the dimerization of the ligand-bound form of NE1028 (PDB code: 3TNJ). The presumably correct biological assembly was ranked second with 0.248 CSS and a decrease of 778 Å^2^ in ASA according to the PISA server. This example shows that neglecting the evolutionary information and looking only at the numerical scores from the prediction servers, it would be very easy to choose a wrong assembly.

Universal stress proteins Rv2623 from *M. tuberculosis*, TTH0350 from *T. thermophilus* HB8, AF1760 from *A. fulgidus* DSM 4304, PMI1202 from *P. mirabilis*, and PA1789 from *P. aeruginosa* are all tandem fusion proteins containing both ‘subunits’ of the probable ‘dimer’ in one polypeptide chain. These fusion proteins form another dimer of tandem ‘dimers’, forming a complex containing four USP domains in total (henceforth called type 2, Fig. 5C). The interactions between the two tandem USP repeats in these proteins are essentially identical to the pattern seen in NE1028 (Fig. 5A). When a single protein chain of the fusion proteins are superimposed on a type 1 dimer, the RMSD for the Cα atoms average 3.4 Å (full dimers superimpose with RMSD values between 0.5 and 4.5 Å). Only small shifts are observed in the positions of α-helices and loops.

**Figure 6:**
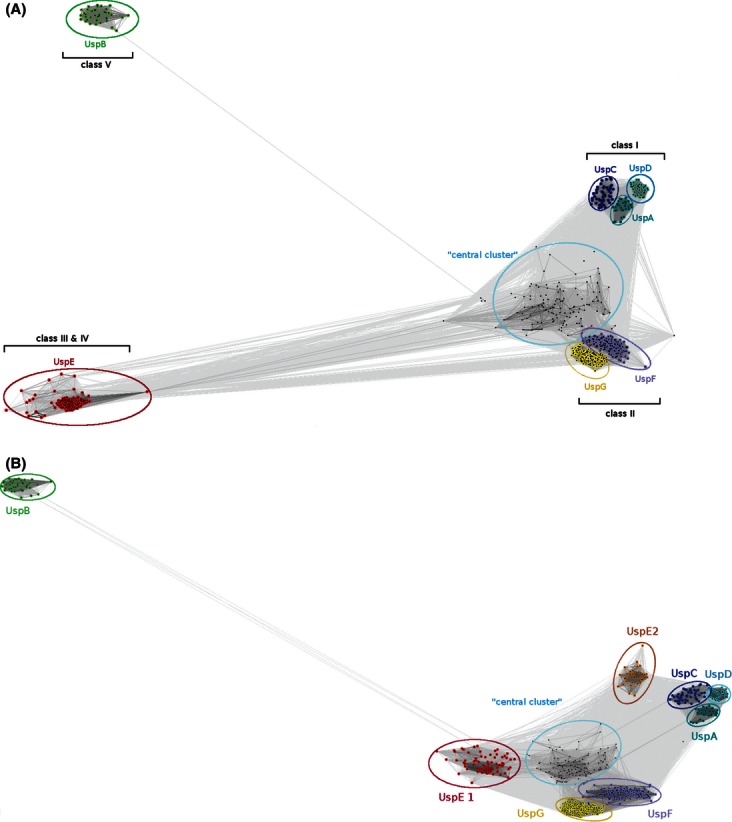
Clustering analysis results. (A) Clustering analysis results with full-length UspE. B shows clustering results with each UspE protein divided into separate domains (UspE1 and UspE2) and treated separately. Each USP family is presented in different colors and labeled.

**Figure 7:**
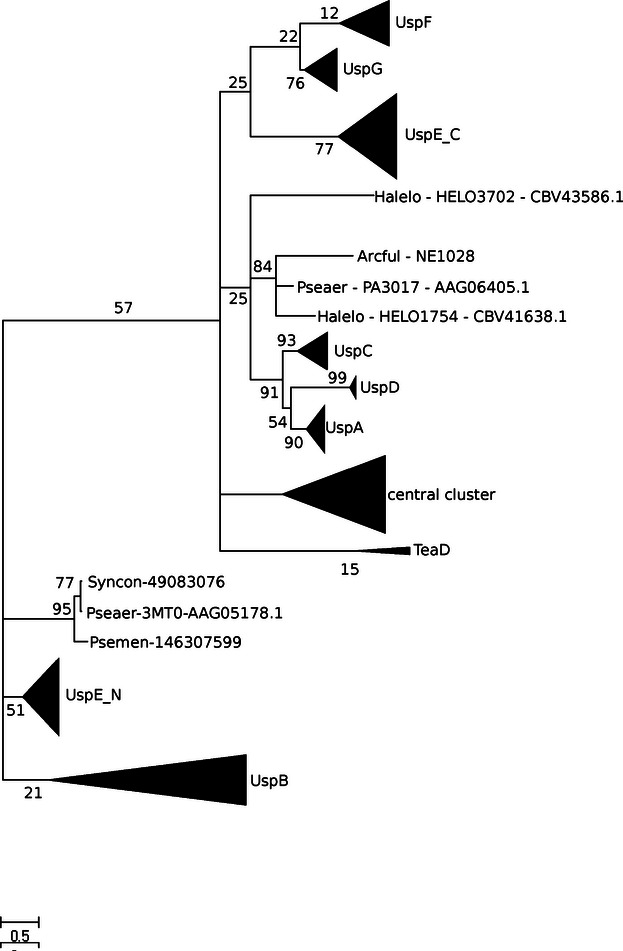
Cladogram depicting USP protein families grouping.

There are two special cases of the type 2 dimers in our data set; namely protein PA1789 from *P. aeruginosa* PAO1 and protein AF1760 from *A. fulgidus* DSM 4304, which are probably biologically relevant as monomers. The biological assembly software PISA also predicts it to be a monomer: attempts to predict biological oligomerization state result in very unusual assemblies with a CSS of 0, which is less than the CSS for monomer—Cl^−^ ion interactions of 0.0176. The *P. aeruginosa* PA1789 protein forms a degenerated pseudo-dimer. In this assembly, the C-terminal part of the protein contains a typical USP domain while the N-terminal one is its degenerated version of the domain, which lacks the α-helix corresponding to residues 274–285 in C-terminal domain. Moreover, it also completely lacks the ATP-binding motif. As shown in Table 1, the cofactor-binding residues come solely from the C-terminal part of the protein and form a slightly degenerated form of the ATP-binding site (G-2X-**G**-9X-G-**S**-T, where the residues in bold are those that differ in PA1789). PISA's best prediction scores for the structures of UspF and UspG from *K. pneumoniae* (i.e., KPN01444) show AA or BB dimers (Fig. 6E) with −*x* + 2, −*x*+*y* + 1, −z + 1/3 the symmetry operation that should be applied to the second interfacing protein chain, rather than AB dimers (symmetry operator: *x*, *y*, *z*) that would look more like other USP dimers (Figs. 6 and 7). This feature makes UspF/G dimers appear to be quite distinctive from other investigated USPs.

### Clustering analysis and evolutionary context

Clustering analysis enabled the division of all the analyzed USPs into groups. Sequences homologous to those of the USP structures determined in this work were retrieved from the NCBI nr (‘non-redundant’) database using the sequences of known USPs, the USP Pfam (PF) families, and all copies of USP from organisms with solved crystal structures. This data set was filtered to obtain a nonredundant input set for the clustering analysis.

We obtained seven clearly separated groups and one additional cluster that is not so well resolved that we called ‘central cluster’ (Fig. 6A). UspB predicted to be an integral membrane (PF10625), as it is considered to be an integral membrane protein it is not a *bona fide* USP protein, thus it was added as an outgroup to facilitate the separation of the nonmembrane USP families. Well-resolved groups correspond mostly to enterobacteria and proteobacteria. The ‘central cluster’ groups together euryarchaeota and bacteria with special characteristics (such as extremophiles and pathogens). This ‘atypical’ cluster is characterized by the unusual adaptation abilities of its members (living in very high temperatures (optimal growth temperature 85°C), as *A. fulgidus* or chemolitoautotrophs like *Nitrosomonas europea*) and forms a special USP group. It includes all copies of USP proteins from TB-causing agent *M. tuberculosis* (apart from the tandem USP Rv2623, which groups together with other UspEs), the function of which is still not known, although predicted to be redundant (Hingley-Wilson et al. 2010), what would at least partially explain their high sequence similarity and clustering within the same group. Unlike USPs from *M. tuberculosis* copies of other pathogenic bacterium, *K. pneumoniae* are distributed among various groups (UspA, UspC, UspG, or UspF), thus may be less functionally redundant; however, there are no experimental data that would confirm or abolish this prediction. Central cluster also contains almost all USPs with 3D structures determined (apart from UspEs). To check of the central cluster is further divided into smaller subgroups, we performed reclustering of only this group. The results of this test were negative; no new subgroups were formed independently on the method/algorithm used for clustering.

UspA, UspC, and UspD form well-defined clusters on their own and cluster into one group. These families were previously described by Nachin et al. (Nachin et al. 2008) as class I USPs. The UspG and UspF clusters are located closer to the ‘central cluster’, and together constitute class II of USPs and are referred to by Nachin and coworkers as UspFG. This class is clearly separated from class I in our clustering analysis (Fig. 6A). The fusion proteins in the UspE group form a well-separated cluster as well. When cut into domains, UspE domain 1 and UspE domain 2, which according to Nachin fall into class III and IV, respectively, in clustering analysis also form two distinct groups as shown on the Fig. 6B. They group USPs from marine organisms, especially green sulfur bacteria adapted to a narrow range of energy-limited conditions and inhabiting the obscure oceanic depths. UspB cluster is named class V.

In 2005 Nachin et al. (2005) concluded that UspD potentially takes part in intracellular iron-level regulation in *E. coli*. Nachin et al. also postulated that UspC does not take part in either stress resistance or iron metabolism, but is essential for motility. They showed that UspE and UspC knockout strains were devoid of flagella, so apparently UspE and UspC are necessary for proper flagella formation. In 2009, Heermann et al. (2009) suggested that UspC scaffolds the KdpD/KdpE signaling cascade of *E. coli* under high-salt conditions. In 2007, Liu et al. (2007) showed that the *uspA* gene from *Salmonella typhimurium* LT2 is induced during metabolic, oxidative, and temperature stress. Another study by Gustavsson et al. (2002) shows that deletion or mutation of any one of *uspA*, *C*, *D,* or *uspE* causes sensitivity to ultraviolet (UV) light. Interestingly, there is no additive effect on UV sensitivity after mutation of more than one *usp* gene. This observation suggests that class I USP paralogs either relate cooperatively or are redundant, and most likely are part of the same functional pathway. These studies show how wide the spectrum of roles played by universal stress protein families is, even within such a tight cluster as class I.

In our analysis, the following proteins of known structure fall into the UspA cluster: HI0815 from *H.* influenzae, KPN03860 from *K. pneumoniae*, PMI3009 from *P. mirabilis*. The UspA cluster was postulated by Aravind et al. (2002) to have evolved from UspFG-like ATPase-binding proteins and then to have further evolved to lose their ATPase activity and nucleotide-binding properties. The UspC cluster contains only one protein with a structure in our data set, namely KPN02391 from *K. pneumoniae*. Postulated to be the most ancestral of all USP families, the UspF/G cluster contains proteins from *K. pneumoniae*—KPN01444 (PDB codes: 3FDX, 3FH0), KPN00652, KPN01588, KPN00789, and most copies of USPs from *P. mirabilis* (bacterium involved in the 90% of all *Proteus* infections in humans, found in kidney stones, and reinitiating kidney infections after antibiotic treatment)—PMI1006, PMI1449, PMI1451, PMI1611, PMI1613, and PMI1954.

UspE, as previously discussed in the literature, is a tandem USP, which most likely evolved from a gene duplication event. UspE contains two separate USP domains, previously described as UspE domain 1 and UspE domain 2. When UspE proteins are split apart and treated separately, the UspE2 domain is more closely related to UspFG as is clearly visible on both the clustering analysis (Fig. 6B) and the reconstructed cladogram (Fig. 7), while UspE1 groups closer to class I USP proteins (UspACD). A good example of an UspE protein is the Rv2623 protein from *M. tuberculosis* (PDB code: 3CIS, Fig. 5C), which superimposes well with the type 1 dimer structures presented in Fig. 5D. The Rv2623 protein is composed of two USP monomers which share 26% sequence identity with one another. Both the N- and C-terminal domains of each monomer contain an ATP-binding motif, and in both cases, a ligand is bound in the structure. Protein TTH0350 from *T. thermophilus* HB8 (PDB code: 3AB7/8) is also an UspE comprising two USP domains, domains 1 (1–152) and 2 (153–268). In TTH0350, the primary structure of domain 1 is 32% identical to that of domain 2. Unlike that of Rv2623, the C-terminal domain of TTH0350 seems to lack the consensus ATP-binding motif. It suggests a possible duplication accompanied by speciation, which resulted in the degeneration of the ATP-binding motif (G-2 x-G-9x-G-(S/T)) in the C-terminal domain. Moreover, the dimer structure of proteins presented in Fig. 5D corresponds well to the monomer of the fusion protein from panel 5C, which means that monomeric proteins most probably adapt the conformation of tandem USPs.

**Figure 8:**
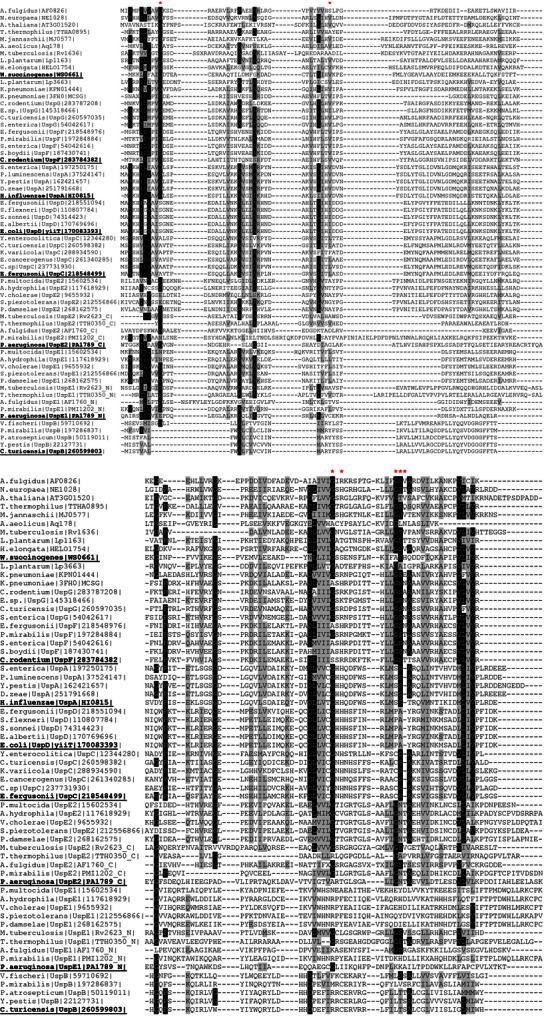
Multiple sequence alignment (MSA) of USP proteins. This figure presents MSA of selected representatives of each family. Invariant and strongly conserved residues are highlighted in black and gray, respectively. Residues interacting with the ligand are marked with asterisk (*).

The division of groups in our cladogram reconstruction (Fig. 7) supports the results obtained from sequence clustering (Fig. 6) and reflects the same grouping as the simple schematic cladogram presented by Gustavsson et al. (2002) and Kvint et al. (2003). UspF, UspG, and UspE2 form very well-resolved branches and group together. As previously mentioned, UspF and UspG form a more closely related group and can be treated as a separate class II, which is evolutionarily closer to class III (UspE) than any other group. Another well-defined branch group is class I containing UspA, UspC, and UspD proteins in close vicinity to which there is a NE1028-like group containing proteins from *P. aeruginosa* (PA3017) and *H. elongate* (HELO1754). As expected, UspB members form a separate well-defined branch distantly related to most other USP families. They have higher similarity to the UspE1 branch and the group represented by PA1789 from *P. aeruginosa* (PDB code: 3MT0), which as mentioned in the section describing dimerization patterns, constitute a unique set of USPs. The central cluster clearly visible on the sequence clustering analysis also constitutes a separate branch on the cladogram. Significant sequential differences between these groups and all traditional USP families are clearly visible on the multiple sequence alignment (MSA) shown on Fig. 8.

In their study of class I aminoacyl-tRNA synthetases (Aravind et al. 2002), Aravind and coworkers conclude that ATP-binding USP domains belong to the HUP domain superfamily, which comprises HIGH-signature proteins, USP-like domains, and PP-ATPases. The results of their analysis, in particular the phyletic distribution of the superfamily, show that this is an ancient domain that already underwent strong diversification in the RNA world. It was suggested that UspFG-like ATPases-binding proteins arose earlier in the evolution than UspA-like ones. Moreover, UspAs underwent further evolutionary events and lost their nucleotide-binding ability or possible ATPase activity, perhaps leading to a wide variety of new or modified functions.

Summarizing our study pinpoints how important evolutionary insight is for the prediction of potential ligand selection and binding, as well as prediction of physiologically relevant biological assemblies. Analysis of other evolutionary-related proteins may also help in functional studies like potential binging mechanism or interaction forming interface predictions. Although we were unable to shade more strictly functional light onto the studied data set, we were able to group collected USPs into specific families (based on their evolutionary similarity). That will help in future to extrapolate the potential functional information (if available for any of the cluster's member) onto other USP proteins that belong to the same group and hopefully speed up experimental testing to confirm their biological function. The results of our analysis show that it is impossible to classify all USP proteins crystallized to date (with couple of exceptions mentioned above) to previously defined families. The reported data shows that four newly determined structures of USPs, together with other crystal structures constitute a separate USP cluster/branch and group together with USPs from extremophilic and pathogenic organisms. Perhaps they form a separate group of USP proteins because of the special adaptation properties of the organisms in which they are found, many of which inhabit extreme environments. Nonetheless, many of the USPs with crystal structures determined fall into particular families as shown in Table 1. As demonstrated by Zarembinski and coworkers (Zarembinski et al. 1998) in the example of *M. jannaschii* MJ0577 (PDB code: 1MJH), protein-structure-based assignment of putative function is indeed possible, especially in larger scale analyses. Of course, in-depth biochemical studies are still necessary to confirm the function; however, the design of these experiments may be guided by bioinformatics results. This analysis stresses out the importance of the structure genomics (SG) initiatives, which as shown in Table 1, provided over 75% of USP crystal structures solved so far, in this way advancing our knowledge about this still poorly characterized superfamily of proteins. Moreover, this work provides summary of structural and bioinformatics analysis of all universal stress protein structures determined and deposited in Protein Data Bank to date.

## Accession numbers

The structures of the archaeal USP AF0826 from *A. fulgidus* have been deposited in the Protein Data Bank under the accession codes 3DLO (*apo*-form) and 3QTB (bound with dAMP), while the structures of the bacterial USP NE1028 from *N. europaea* are deposited with codes 2PFS (*apo*-form) and 3TNJ (bound with AMP).
